# A Facile Fabrication of Alginate Microbubbles Using a Gas Foaming Reaction

**DOI:** 10.3390/molecules18089594

**Published:** 2013-08-12

**Authors:** Keng-Shiang Huang, Yung-Sheng Lin, Wan-Ru Chang, Yi-Ling Wang, Chih-Hui Yang

**Affiliations:** 1 The School of Chinese Medicine for Post-Baccalaureate, I-Shou University, Kaohsiung 82445, Taiwan; E-Mail: huangks@isu.edu.tw; 2 Department of Applied Cosmetology and Master Program of Cosmetic Science, Hungkuang University, Taichung 43302, Taiwan; E-Mail: linys@sunrise.hk.edu.tw; 3 Department of Biological Science and Technology, I-Shou University, Kaohsiung 82445, Taiwan; E-Mails: dale1511@hotmail.com (W.-R.C.); callingsummer@hotmail.com (Y.-L.W.)

**Keywords:** microbubbles, alginate, gas, particle

## Abstract

Microbubble particles have been extensively utilized as temporal templates for various biomedical applications. This study proposes a facile strategy to obtain microbubble-containing alginate particles (*i.e.*, microbubbles inside alginate gel particles, called alginate microbubbles). The chemical reaction of sodium bicarbonate and hydrogen peroxide to produce gaseous carbon dioxide and oxygen was utilized to form microbubbles within alginate particles. Uniform alginate particles were obtained by a stable needle-based droplet formation process. Kinetic reaction of gas formation was monitored for 2% alginate particles. The gas formation increased with the concentrations of sodium bicarbonate (1–5 wt%) and hydrogen peroxide (0–36.5 wt%).

## 1. Introduction

Porous biodegradable polymer substrates have extensive biomedical applications [1]. There has been a drastic increase in the use of microbubbles as vehicles for ultrasound-mediated imaging and targeted drug delivery [2,3,4,5,6,7]. Typical methods to fabricate porous structures include gas foaming [8], air pressure-driven injection [9], porogen elimination [10], emulsion/freeze drying [11,12], expansion in supercritical fluids [13], 3D ink-jet printing [14,15], *etc*. Some novel strategies to make microbubbles have been reviewed [16,17]. Among these methods, gas foaming based on a chemical reaction is one of the most simple and straightforward routes to form pores within a particle. It needs no extra equipment or instruments for porous structure generation. Besides being simple, low-cost, and easy to operate, chemical reactions have the added advantage of being effective in making gas pores [8].

Alginate is a natural polysaccharide derived from marine brown algae that finds numerous applications in diverse areas [18,19,20]. The two major fields of alginate applications are as biomedical devices for drug delivery and tissue engineering and as adsorbent materials for elimination of heavy metals and organic pollutants in water [21]. For these uses, exposed surface area is a crucial issue to determine its success. A high surface area will facilitate adsorption of water pollutants, but only suitable pore morphology can favor biomedical applications such as cell colonization. Alginate particles with porous inner structures have wide applications ranging from pharmaceuticals to foods and the cosmetic industry [1,16,22]. They have strong potential for molecular imaging, drug delivery, gene therapy, sonothrombolysis, and therapeutic treatment of antimicrobial films [23]. Calcium chloride-crosslinked gel-type alginate has a high viscosity, being capable of holding microbubbles for a long time [24]. These porous alginate particles can be applied as a contrast agent to enhance the ultrasound echo from the stomach and intestine through oral administration [24]. Furthermore, porous alginate particles can combine chemotherapeutic and imaging agents for ultrasound-mediated drug delivery because contrast-enhanced imaging provides for precise energy deposition [25].

Based on our previous works on the manufacture of uniform alginate particles [26,27,28,29], this work further develops a facile method to fabricate microbubble-containing alginate particles. The porous alginate particles (alginate microbubbles) prepared under different gas forming conditions were investigated and characterized. The manufactured alginate microbubbles provide great promise for biomedical applications such as chemotherapeutics and imaging.

## 2. Results and Discussion

### 2.1. Ca-Alginate Microbubbles

**Figure 1:**
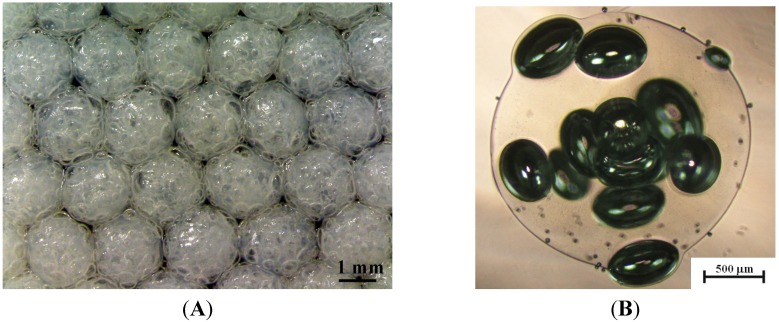
Photo images of porous alginate particles. (**A**) Uniform microparticle distribution with an average diameter of 2.25 ± 0.11 mm and (**B**) Close-up view of a single microparticle with gas bubbles inside.

The stable needle-based droplet formation ensured a uniform particle size distribution. Figure 1 shows the collected Ca-alginate microbubbles in the receiving collector under the condition of 2% sodium bicarbonate and 18.25% hydrogen peroxide. As indicated in the pictures, the Ca-alginate particles are uniform in both morphology and size, and the average diameter is 2.12 ± 0.15 mm (Figure 1A). In a close view (Figure 1B), gas development was randomly distributed within the alginate particle. The diameter of microbubbles reached several tens or hundreds micrometers and these pore sizes favor applications in cell colonization or metabolic waste removal, *etc.* [21]. The pore-closed surface may be due to the high viscosity of gel-type alginate that holds microbubbles entrapped in the particle [24].

### 2.2. Microbubble Evolution

**Figure 2:**
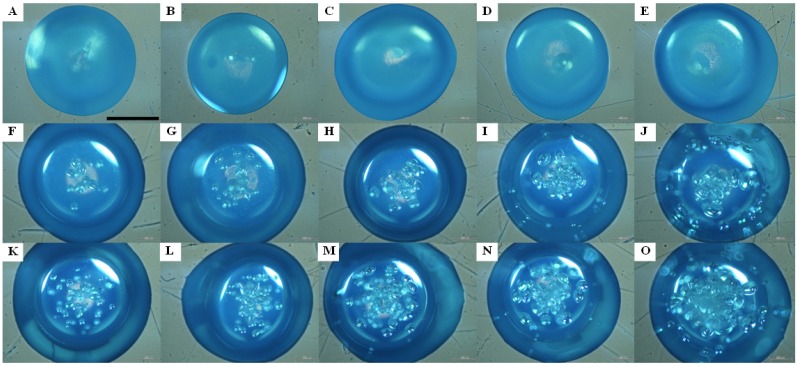
Photo images of the kinetics of gas formation in a single alginate microparticle. The concentration of NaHCO_3_ is fixed at 1% and the H_2_O_2_ concentrations are 0% (**A**–**E**), 18.25% (**F**–**J**), and 36.5% (**K**–**O**). The gas formation time are 30 seconds (**A**, **F**, **K**), 1 minute (**B**, **G**, **L**), 3 minutes (**C**, **H**, **M**), 5 min (**D**, **I**, **N**), and 10 min (**E**, **J**, **O**). Scale bar is 1 mm.

Figure 2 indicates microbubble evolution in a kinetic process. The sodium bicarbonate is fixed at 1%, and three hydrogen peroxide concentrations (0%, 18.25%, 36.5%) are used for comparison. The predetermined five observation times are 30 seconds, 1 min, 3 min, 5 min, and 10 min, respectively. The photo image results show that the size and number of microbubbles increases with reaction time. Furthermore, microbubble development within an alginate particle is more conspicuous at a higher hydrogen peroxide concentration. The gas evolution after ten minutes is not significant (data not shown). Therefore, we collected porous alginate after alginate was immersed in hydrogen peroxide solution for ten minutes in the following study.

**Figure 3:**
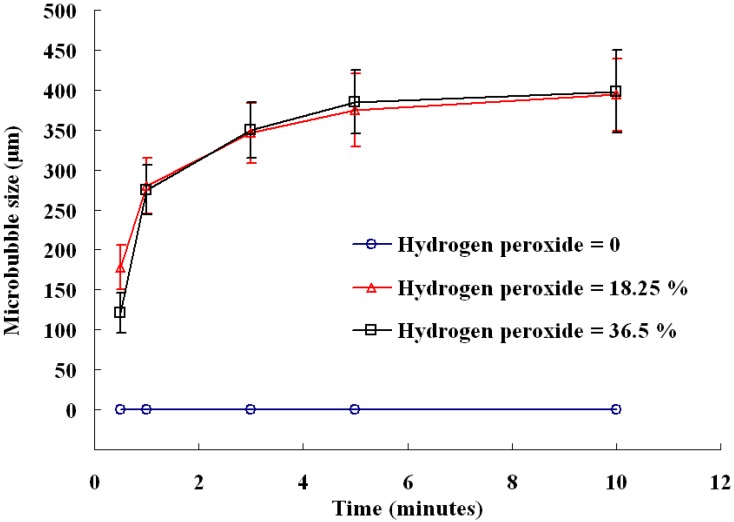
Evolution of microbubble size in alginate particles under the conditions of Figure 2.

Figure 3 indicates the average size of microbubbles in the gas formation process. Results show that the there is no microbubble formation in the absence of hydrogen peroxide. Both the sodium bicarbonate and hydrogen peroxide are needed to produce the oxidative/reductive reaction that generates the gas. The size of microbubbles can reach about 400 μm. Furthermore, there is little difference between 18.25% and 36.5% hydrogen peroxide except in the early reaction stages. A smaller microbubbles size is formed in the early reaction phase for a higher hydrogen peroxide concentration. Due to the larger number of reactant molecules in 36.5% hydrogen peroxide, the number of reaction nuclei for each gas pore is larger. This results in a smaller microbubble size for a fixed sodium bicarbonate concentration.

### 2.3. Influence of Reactant Concentration

**Figure 4:**
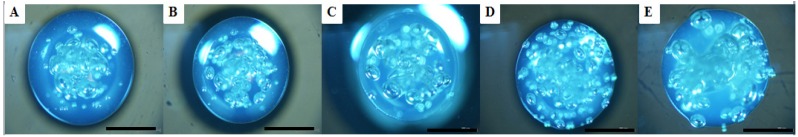
Photo images of a single alginate microparticle prepared by a fixed 36.5% H_2_O_2_ and different NaHCO_3_ concentrations: (**A**) 1%, (**B**) 2%, (**C**) 3%, (**D**) 4%, (**E**) 5%. All scale bars are 1 mm.

**Figure 5:**
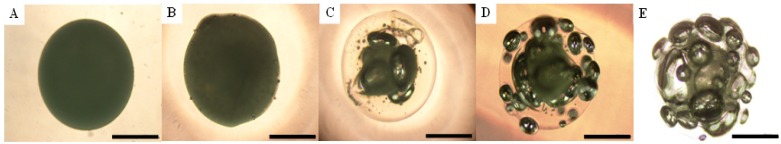
Photo images of a single alginate microparticle prepared by a fixed 5% NaHCO_3_ and different H_2_O_2_ concentrations: (**A**) 0%, (**B**) 9.12%, (**C**) 18.25%, (**D**) 27.37%, (**E**) 36.5%. All scale bars are 1 mm.

Figure 4 and Figure 5 investigate effects of sodium bicarbonate and hydrogen peroxide on the formation of microbubbles, respectively. To enhance the difference, the concentration of hydrogen peroxide is fixed at 36.5% in Figure 4, while sodium bicarbonate concentration is fixed at 5% in Figure 5. Results reveal that gas formation increases with the concentration of sodium bicarbonate (1%–5% in Figure 4) and hydrogen peroxide (0%–36.5% in Figure 5). Microbubble development within an alginate particle showed an enhancement for a high reactant concentration for both sodium bicarbonate and hydrogen peroxide. There is almost no gas formation in the absence of or with and 9.12% hydrogen peroxide (Figure 5).

Due to the short diffusion path, the outer region of a particles showed an obvious gas formation, especially for a high reactant concentration. Most conventional contrast agents are vulnerable and lack stability [11]. Microbubbles are easily destroyed during the ultrasound diagnostic process. To have good detection quality, some constraints should be adopted, such as continuously infusing microbubbles, or lowering the ultrasound power, frame rate, and scan line density. Microbubbles composed of alginate have the advantage of eliminating these mentioned inconveniences. Alginate is a viscous hydrogel capable of holding microbubbles [24] for a good contrast agent efficacy. Besides, microbubble alginates can also carry chemotherapeutic agents for drug delivery applications.

Some novel microbubble preparation technologies are established. Microfluidic devices or other hydrodynamic atomization techniques can offer great control of microbubble size and polydispersity [16,17,30]. However these methods are usually cost-prohibitive, needing an extra gas source and equipment/instruments for microbubble generation. Besides, the production rate of microbubble particles is limited. Overcoming these inconveniences, the simple sodium bicarbonate-based gas formation proposed in this study offers the facile strategy to prepare microbubble particles. It has advantages of being simple, low-cost, and easy to operate. The gas formation reaction thus provides an alternative practical method in making gas pores [8].

## 3. Experimental

### 3.1. Materials

Alginic acid sodium salt (Na-alginate, brown algae with a viscosity 250 cP in 2% (w/v) solution at 25 °C) and hydrogen peroxide (36.5 wt%) were purchased from Sigma Chemical Co. (St. Louis, MO, USA). Calcium chloride and sodium bicarbonate (dehydrate, granular) were obtained from J.T. Baker Chemical Company (Phillipsburg, NJ, USA). All chemicals and solvents were of analytical reagent grade.

### 3.2. Preparation of Ca-Alginate Particles

**Figure 6:**
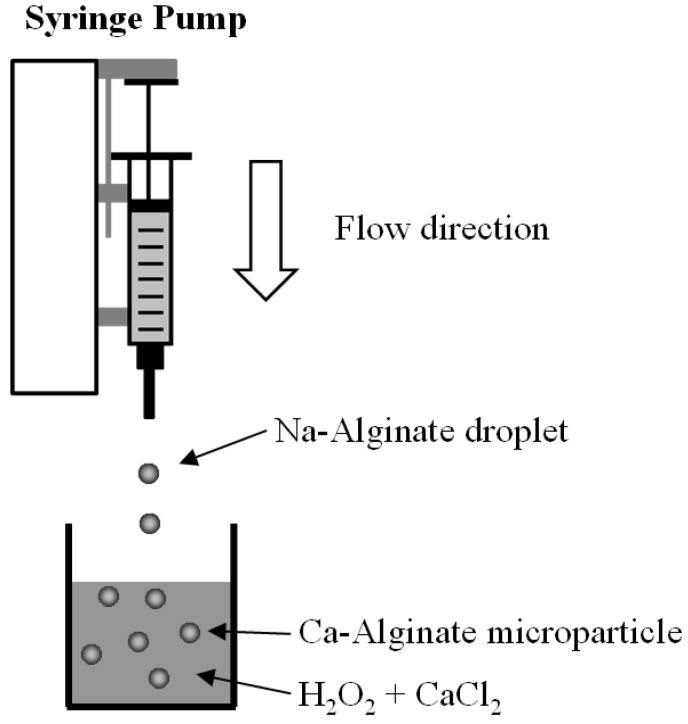
Schematic drawing of the experimental setup.

As shown in Figure 6, Na-alginate solution loaded in a syringe (TERUMOR^®^ Syringe, 3 mL) was extruded from the needle tip (24 G, 0.55 × 25 mm) at a 0.01 mL/min constant rate by a KDS230 syringe pump (KD Scientific Inc., Holliston, MA, USA). To generate microbubbles, sodium bicarbonate must be incorporated homogeneously with the Na-alginate solutions. The pendant Na-alginate solution at the tip of the needle was broken up to form a series of isolable Na-alginate droplets of about 2–3 mm. The liquid in the receiving collector was filled with 25% w/v calcium chloride solution (2.5 grams calcium chloride in 10 mL hydrogen peroxide solution) for gelation. Sodium-alginate droplets are gelled *in situ* by immersion of Ca^2+^ ions for ten minutes, and then finally Ca-alginate particles were collected and characterized.

### 3.3. Preparation of Ca-Alginate Microbubbles

**Figure 7:**
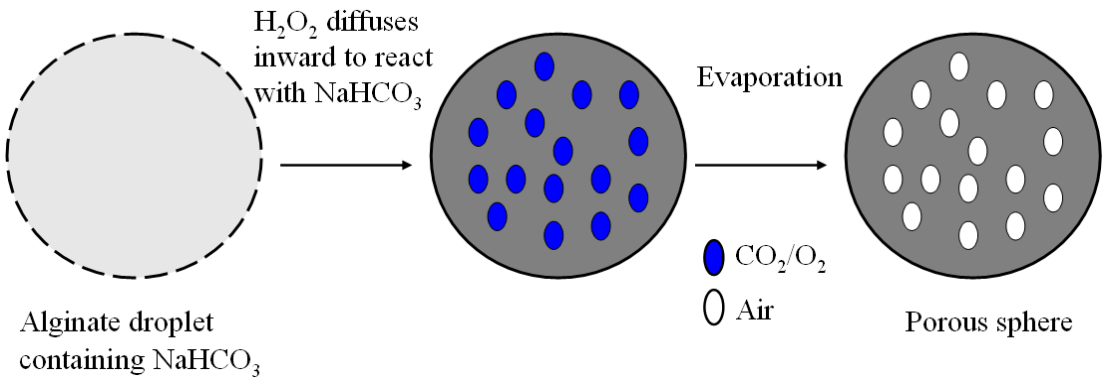
Schematic picture of structure evolution within a microparticle to form pores.

The gas formation within Ca-alginate particles can be illustrated by Figure 7. First of all, sodium bicarbonate was incorporated homogeneously in Na-alginate solutions. When the Na-alginate droplets were immersed in the receiving collector, hydrogen peroxide diffused inward into the droplet to react with sodium bicarbonate.

Then carbon dioxide and oxygen gas can be generated *in situ* by an oxidative/reductive reaction between sodium bicarbonate and hydrogen peroxide. This process can be described by the following reactions [31]:

NaHCO_3_ + H_2_O_2_ ↔ NaHCO_4_·H_2_O

NaHCO_4_·H_2_O → Na_2_CO_3_·1.5 H_2_O_2_ + CO_2_ + 1.5 H_2_O + 0.25 O_2_

After gas exchange with the surrounding air, the generated CO_2_ and O_2_ diffused out, and the microbubbles within the particle were finally filled with air.

### 3.4. Characterization

An optical microscope system (TE2000U, Nikon, Lewisville, TX, USA) and a digital camera (Evolution color VF, Media Cybernetics, Silver Spring, MD, USA) were utilized to observe the morphology of the collected particles. To ensure statistical representation, more than 50 particles were analyzed. The size of microbubbles was obtained from their photomicrographs and expressed as mean ± SD (standard deviation).

## 4. Conclusions

Alginate microbubbles have great potential to combine chemotherapeutic and imaging agents for ultrasound-mediated drug delivery applications. Providing a cost-effective approach, this study successfully developed a sodium bicarbonate/hydrogen peroxide-based chemical reaction to generate porous alginate particles. The gas formation increased with the reactant concentration, and the gas reaction evolved in ten minutes for an alginate particle of 2 mm in diameter. This finding provides a facile strategy to prepare microbubble particles. This proposed method has the advantages of easy operation, low cost, and effective pore formation.

## References

[1] Kim T.K., Yoon J.J., Lee D.S., Park T.G. (2006). Gas foamed open porous biodegradable polymeric microspheres. Biomaterials.

[2] Dressaire E., Bee R., Bell D.C., Lips A., Stone H.A. (2008). Interfacial polygonal nanopatterning of stable microbubbles. Science.

[3] Capece S., Chiessi E., Cavalli R., Giustetto P., Grishenkov D., Paradossi G. (2013). A general strategy for obtaining biodegradable polymer shelled microbubbles as theranostic devices. Chem. Commun..

[4] Hosny N.A., Mohamedi G., Rademeyer P., Owen J., Wu Y., Tang M.X., Eckersley R.J., Stride E., Kuimova M.K. (2013). Mapping microbubble viscosity using fluorescence lifetime imaging of molecular rotors. Proc. Natl. Acad. Sci. USA.

[5] Geers B., de Wever O., Demeester J., Bracke M., de Smedt S.C., Lentacker I. (2013). Targeted liposome-loaded microbubbles for cell-specific ultrasound-triggered drug delivery. Small.

[6] Noble M.L., Kuhr C.S., Graves S.S., Loeb K.R., Sun S.S., Keilman G.W., Morrison K.P., Paun M., Storb R.F., Miao C.H. (2013). Ultrasound-targeted microbubble destruction-mediated gene delivery into canine livers. Mol. Ther..

[7] Villa R., Cerroni B., Viganò L., Margheritelli S., Abolafio G., Oddo L., Paradossi G., Zaffaroni N. (2013). Targeted doxorubicin delivery by chitosan-galactosylated modified polymer microbubbles to hepatocarcinoma cells. Colloids Surf. B Biointerfaces.

[8] Bae S.E., Son J.S., Park K., Han D.K. (2009). Fabrication of covered porous PLGA microspheres using hydrogen peroxide for controlled drug delivery and regenerative medicine. J. Control. Release.

[9] Wang X.L., Li X., Stride E., Huang J., Edirisinghe M., Schroeder C., Best S., Cameron R., Waller D., Donald A. (2010). Novel preparation and characterization of porous alginate films. Carbohyd. Polym..

[10] Chevalier E., Chulia D., Pouget C., Viana M. (2008). Fabrication of porous substrates: A review of processes using pore forming agents in the biomaterial field. J. Pharm. Sci..

[11] Cui W., Bei J., Wang S., Zhi G., Zhao Y., Zhou X., Zhang H., Xu Y. (2005). Preparation and evaluation of poly(L-lactide-co-glycolide) (PLGA) microbubbles as a contrast agent for myocardial contrast echocardiography. J. Biomed. Mater. Res. B Appl. Biomater..

[12] Zhang H., Ju X.J., Xie R., Cheng C.J., Ren P.W., Chu L.Y. (2009). A microfluidic approach to fabricate monodisperse hollow or porous poly(HEMA-MMA) microspheres using single emulsions as templates. J. Colloid Interface Sci..

[13] Butler R., Davies C.M., Cooper A.I. (2001). Emulsion templating using high internal phase supercritical fluid emulsions. Adv. Mater..

[14] Yang S., Leong K.F., Du Z., Chua C.K. (2002). The design of scaffolds for use in tissue engineering. Part II. Rapid prototyping techniques. Tissue Eng..

[15] Tsang V.L., Bhatia S.N. (2004). Three-dimensional tissue fabrication. Adv. Drug Deliv. Rev..

[16] Stride E., Edirisinghe M. (2008). Novel microbubble preparation technologies. Soft Matter.

[17] Stride E., Edirisinghe M. (2009). Novel preparation techniques for controlling microbubble uniformity: A comparison. Med. Biol. Eng. Comput..

[18] Coppi G., Iannuccelli V. (2009). Alginate/chitosan microparticles for tamoxifen delivery to the lymphatic system. Int. J. Pharm..

[19] Chen C.C., Fang C.L., Al-Suwayeh S.A., Leu Y.L., Fang J.Y. (2011). Transdermal delivery of selegiline from alginate-Pluronic composite thermogels. Int. J. Pharm..

[20] Balaure P.C., Andronescu E., Grumezescu A.M., Ficai A., Huang K.S., Yang C.H., Chifiriuc C.M., Lin Y.S. (2013). Fabrication, characterization and *in vitro* profile based interaction with eukaryotic and prokaryotic cells of alginate-chitosan-silica biocomposite. Int. J. Pharm..

[21] Barbetta A., Barigelli E., Dentini M. (2009). Porous alginate hydrogels: Synthetic methods for tailoring the porous texture. Biomacromolecules.

[22] Wan J., Bick A., Sullivan M., Stone H.A. (2008). Controllable microfluidic production of microbubbles in water-in-oil emulsions and the formation of porous microparticles. Adv. Mater..

[23] Cavalieri F., Zhou M., Tortora M., Lucilla B., Ashokkumar M. (2012). Methods of preparation of multifunctional microbubbles and their *in vitro*/*in vivo* assessment of stability, functional and structural properties. Curr. Pharm. Des..

[24] Soetanto K., Chan M., Okujima M. (1996). Effect of calcium chloride on sodium alginate microbubbles as ultrasound contrast agent. Jpn. J. Appl. Phys..

[25] Sridhar-Keralapura M., Thirumalai S., Mobed-Miremadi M. (2013). Structural changes and imaging signatures of acoustically sensitive microcapsules under ultrasound. Ultrasonics.

[26] Huang K.S., Yang C.H., Lin Y.S., Wang C.Y., Lu K., Chang Y.F., Wang Y.L. (2011). Electrostatic droplets assisted synthesis of alginate microcapsules. Drug Deliv. Transl. Res..

[27] Huang K.S., Lin Y.S., Yang C.H., Tsai C.W., Hsu M.Y. (2011). *In situ* synthesis of twin monodispersed alginate microparticles. Soft Matter.

[28] Wang C.Y., Yang C.H., Lin Y.S., Chen C.H., Huang K.S. (2012). Anti-inflammatory effect with high intensity focused ultrasound-mediated pulsatile delivery of diclofenac. Biomaterials.

[29] Lin Y.S., Yang C.H., Hsu Y.Y., Hsieh C.L. (2013). Microfluidic synthesis of tail-shaped alginate microparticles using slow sedimentation. Electrophoresis.

[30] Gong X., Wen W., Sheng P. (2009). Microfluidic fabrication of porous polymer microspheres: Dual reactions in single droplets. Langmuir.

[31] Firsova T.P., Sokol V.I., Bakulina V.M., Stasevich N.N. (1968). Reaction of sodium bicarbonate with hydrogen peroxide and some properties of the compound Na_2_CO_3_ 1.5 H_2_O_2_. Russ. Chem. Bull..

